# Research statistics in Atopic Eczema: what disease is this?

**DOI:** 10.1186/1824-7288-38-26

**Published:** 2012-06-09

**Authors:** Kam-Lun Ellis Hon, Vivien Yong, Ting-Fan Leung

**Affiliations:** 1 Department of Pediatrics, Prince of Wales Hospital, The Chinese University of Hong Kong, Clinical Science Building, 6/F, Shatin, Hong Kong; 2 Faculty of Medicine, University of Auckland, Auckland, New Zealand

**Keywords:** Atopic dermatitis, Eczema, ISI, Impact Factors, PubMed

## Abstract

****Background**:**

Atopic eczema is a common and distressing disease. This study aims to review PubMed indexed research statistics on atopic eczema over a-10 year period to investigate the clinical relevance and research interest about this disease.

****Methods**:**

PubMed (a service of the U.S. National Library of Medicine) was searched for the terms “atopic dermatitis” and “eczema”, with limits activated (Humans, Clinical Trial, Meta-Analysis, Randomized Controlled Trial, English, published in the last 10 years), and editorials, letters, practice guidelines, reviews, and animal studies excluded. Journal impact factor (IF) is in accordance with Journal Citation Report (JCR) 2009, a product of Thomson ISI (Institute for Scientific Information).

****Results**:**

A total of 890 articles were retrieved. Taking out publications that were irrelevant and those without an impact factor, 729 articles were obtained. These articles were grouped into dermatology (n = 337, mean IF: 3.01), allergy/immunology (n = 215, mean IF: 4.89), pediatrics (n = 118, mean IF: 2.53) and miscellaneous subject categories (n = 142, mean IF: 5.10). The impact factors were highest in the miscellaneous category (*p* = 0.0001), which includes such prestigious journals as the New England journal of Medicine (n = 1, IF: 47.05), the Lancet (n = 4, IF: 30.76) and BMJ (n = 6, IF: 13.66). There was no publication in any family medicine or general practice journal. The British Journal of Dermatology (n = 78), Pediatric Allergy and Immunology (n = 49) and Journal of Allergy and Clinical Immunology (n = 46) had the highest number of publications on the subject. Atopic eczema ranked higher in impact factors in allergy/immunology although more publications appeared in the dermatology category.

****Conclusions**:**

Atopic eczema is a multidisciplinary disease. Its clinical relevance and research interests are definitely beyond that of a mere cutaneous disease. Investigators may consider allergy/immunology and miscellaneous journal categories for higher impact of their research.

## **Introduction**

Childhood eczema is a distressing disease associated with atopy [1-6]. Disease onset is usually before 5 years of age in the majority of patients [2,7]. The presence of atopy, according to the theory of Atopic March, implies that young children with eczema may develop airway allergy such as asthma or allergic rhinitis later in life [5,6,8]. Atopy is defined clinically (personal or family history of eczema, asthma or allergic rhinitis) and by laboratory tests (such as positive skin prick reaction to common food and aeroallergens or elevated serum IgE levels above laboratory reference range for age) [4,9-11]. This study aims to review PubMed-indexed research publications statistics on atopic eczema over a-10 year period to investigate the clinical relevance and research interests about this disease.

## **Methods**

PubMed (a service of the U.S. National Library of Medicine) was searched for the terms “atopic dermatitis” and “eczema”, with limits activated (Humans, Clinical Trial, Meta-Analysis, Randomized Controlled Trial, English, published in the last 10 years), and editorials, letters, practice guidelines, reviews, and animal studies excluded. These limits were set so that the data could be manageable. Journal impact factor (IF) is in accordance with Journal Citation Report (JCR) 2009, a product of Thomson ISI (Institute for Scientific Information). Data was expressed as mean and standard deviation (SD) unless otherwise stated. ANOVA was used to compare means. All comparisons were made two-tailed, and *p*-values less than 0.05 were considered to be statistically significant.

## **Results**

Between 2001 and 2011 (as of April 2011), a total of 890 articles were retrieved. Each article/journal was reviewed for relevancy and for an impact factor by Journal Citation Report JCR 2009. Taking out publications that are irrelevant and those without an impact factors, 729 articles were obtained. They were classified into dermatology, allergy/immunology, pediatrics, and miscellaneous categories (Table 1). The journal Pediatric Dermatology was double-counted in both pediatrics and dermatology, and Paediatric Allergy and Immunology was double-counted in both pediatrics and allergy/immunology. International Journal of Pediatric Otolaryngology, Paediatric and Perinatal Epidemiology, Pediatric Pulmonology, and Pediatric Infectious Disease Journal were double-counted in both pediatrics and miscellaneous. Skin Pharmacology and Physiology and Dermatologic Surgery were double-counted in both dermatology and miscellaneous. FEMS Immunology and Medical Microbiology, International Journal of Immunopathology and Pharmacology, Genes and Immunity and Seminars in Immunopathology were double counted in both allergy/immunology and miscellaneous.

**Table 1:** Number of publications in the three subject categories and their impact factors

**Journal**	**Number of publications**	**Impact factor**
Dermatology	337	
British Journal of Dermatology	78	4.260
Journal of the American Academy of Dermatology	33	4.105
Pediatric Dermatology	16	1.031
Contact Dermatitis	9	3.635
Dermatologic Surgery	1	2.343
Skin Pharmacol Physiol	4	2.117
Acta Dermato-Venereologica	15	3.007
Acta Dermatovenerologica Croatica	3	0.461
American Journal of Clinical Dermatology	4	1.820
Archives of Dermatology	9	4.760
Australasian Journal of Dermatology	2	0.973
Clinical and Experimetnal Dermatology	13	1.550
Cutis	10	1.019
Dermatitis	4	2.264
Dermatologic Therapy	1	1.828
Dermatology	21	2.741
European Journal of Dermatology	5	2.251
Experimental Dermatology	8	3.239
Indian Journal of Dermatol Venereol Leprol	2	0.976
International Journal of Dermatology	12	1.177
Journal of Cutaneous Medicine and Surgery	1	1.096
Journal of Dermatological Science	5	3.713
Journal of Dermatology	11	1.008
Journal of Dermatologic Treatment	24	1.571
Journal der Deutschen Dermatologischen Gesellschaft	5	1.403
Journal of European Acad Dermatol Venereol	20	5.543
Journal of Investigative Dermatology	5	5.543
Photodermatol Photoimmunol Photomed.	8	1.604
Skin Research and Technology	8	1.307
Allergy/Immunology	215	
Pediatric Allergy and Immunology	49	2.676
Journal of Allergy & Clinical Immunology	46	9.165
Allergy	37	6.380
Allergy and Asthma Proceedings	3	1.796
Annals of Allergy Asthma & Immunology	10	2.457
Clinical and Expierimental Allergy	34	4.084
Genes and Immunity	1	4.222
Clinical and Experimental Immunology	1	2.550
Clinical Immunology	2	3.863
International Arch Allergy & Immunology	8	2.542
Journal of Clinical Immunology	1	3.583
Journal of Investigational Allergology & Clinical Immunology	10	1.189
Contact Dermatitis.	9	3.635
FEMS Immunology and Medical Microbiology	1	2.335
International Journal of Immunopathology & Pharmacology	1	3.061
Seminars in Immunopathology	1	9.155
Allergol Immunopathol (Madr).	1	0.630
Pediatrics	118	
Pediatric Allergy and Immunology	49	2.676
Pediatric Dermatology	16	1.031
International Journal of Pediatr Otolaryngol	1	1.148
Paediatr Perinat Epidemiol	2	1.797
Pediatr Infect Disease Journal	1	2.854
Pediatr Pulmonol.	4	1.816
Acta Paediatr	4	1.768
Arch Dis Child	7	2.657
Arch Pediatr Adolesc Medicine	4	4.726
European Journal of Pediatrics	1	1.634
Indian Paediatrics	1	0.962
Journal of Paediatrics and Child Health	1	1.138
Journal of Pediatrics (Rio J)	2	1.382
Journal of Pediatric Gastroenterology & Nutrition	5	2.183
Journal of Pediatric Hematology and Oncology	1	1.022
Journal of Pediatrics	7	4.092
Journal of Tropical Pediatrics	1	1.224
Pediatr Research	2	2.607
Pediatrics	8	4.687
World Journal of Pediatrics	1	0.365

142 publications in miscellaneous category not listed.

These articles were grouped into dermatology (n = 337), allergy/immunology (n = 215), pediatrics (n = 118) and miscellaneous subject categories (n = 142) (Table 2). The impact factors were highest in the miscellaneous category (*p* = 0.0001). There were a number of prestigious journals in the miscellaneous category, which included the New England journal of Medicine (n = 1, IF: 47.05), the Lancet (n = 4, IF: 30.76) and BMJ (n = 6, IF: 13.66).

**Table 2:** Indexed publications with impact factors

**Category**	**Dermatology**	**Allergy**	**Pediatrics**	**Miscellaneous**
Number	337	215	118	142
IF [mean (SD)]*	3.01 (1.47)	4.89 (2.69)	2.53 (1.08)	5.10 (6.55)
95% CI	2.67–3.36	4.46–5.33	1.93–3.11	4.57–5.63
High	5.54	9.17	4.73	47.05
Low	0.46	0.56	0.37	0.51
Median	3.01	4.08	2.68	2.83

*P = 0.0001 by ANOVA.

Eight publications were under psychiatry/psychology: Psychotherapy and Psychosomatics (n = 1), Psychosomatic Medicine (n = 3), The Journal of Behavioral Medicine (n = 1) and Journal of Psychosomatic Research (n = 3). Five publications were under integrative and complementary medicine: Complementary Therapies in Medicine (n = 2), American Journal of Chinese Medicine (n = 2), Journal of Alternative and Complement Medicine (n = 2) and Journal of Ethnopharmacology (n = 1). There was no publication in any family medicine or general practice journal. The British Journal of Dermatology (n = 78), Pediatric Allergy and Immunology (n = 49) and Journal of Allergy and Clinical Immunology (n = 46) had the highest number of publications on the subject. Atopic eczema ranked higher in impact factors in allergy/immunology although more publications appeared in the dermatology category.

## **Discussion**

Childhood eczema is a common chronic relapsing disease [1,2,12] The theory of “atopic march” views eczema as a systemic atopic disease with skin manifestation in early childhood, and subsequent airway manifestations [2,5,8,11]. Our study of more than seven hundred articles published over a ten-year period showed that eczema is a clinical entity with diverse research interests. Although dermatology journals rank top in number, allergy and medicine journals have higher impact factors and reflect higher research interests and priority. There are also publications in the top medical journals with impact factors above 30, such as the New England Journal of Medicine and the Lancet. In many Asian cities, herbal medicine is extensively sought after by many parents and patients in preference to western medicine [13]. The scope is disproportionately represented by only few articles in the complementary medicine category [14]. Eczema contributes a heavy work load for general practitioners. Surprisingly, no publications were represented in the family medicine categories.

The limitations of this study are that many advances in basic research are not represented due to selection bias of only clinical categories. For instance, filaggrin and related genome wide studies are published in top science journals in recent years [15-17]. The number of articles would be doubled if articles in non-English journals were also included, which was especially true for the complementary medicine category [14]. The number of journals included would certainly increase if a longer study period, say 20 years, is included. With the search limits, “case reports” are also included, which tend to be cited less often than randomized controlled trials and meta-analyses. As such, journals that publish mainly or only randomized controlled trials tend to have higher impact factors.

In dermatology, the “brick-and-mortar hypothesis” states the stratum corneum (the outermost layer of the epidermis) normally consists of fully differentiated corneocytes surrounded by a lipid-rich matrix containing cholesterol, free fatty acids, and ceramide; the structure of this matrix closely resembles that of bricks and mortar in a wall. In eczema, lipid metabolism is abnormal, causing a deficiency of ceramide that leads to transepidermal water loss [18-20]. The underlying genetic deficit might be due to null mutation in the filaggrin gene [17]. Treatment of eczema is primarily with emollient and topical steroid/immunomodulating agent usage [2]. On the contrary, the theory of “Atopic March” favors the consideration of eczema as a systemic disease, and indicates that many children with atopic eczema go on to develop asthma and allergic rhinitis as their eczema improves with time [5,6,8]. Nitric oxide (NO) has been shown to be a marker of airway inflammation. Study has shown levels of NO in exhaled breath condensate are higher in children with eczema without asthma, and may indicate a predictive role of exhaled NO for the development of asthma [21]. Atopic eczema has long been considered as a disease primarily driven by the immune system [2]. Eosinophils and monocytes are known for modulating allergic symptoms. Eosinophilia with enhanced eosinophil survival and elevated eosinophil granule proteins have been detected in AD patients. Overactive monocytes increase the production of prostaglandin E and IL-10, which alter the balance between Th1 and Th2 functional responses. This accounts for many atopic features present in eczema patients, including elevated IL-4, 5 and 6 productions by T cell, increased IgE synthesis, reduced IFN-γ production and impaired cell-mediated immune response [2]. The “hygiene hypothesis” further explains the skewing of immune system towards Th2 profile [22]. Initial atopic sensitization is thought to take place in utero where transplacental allergen elicits Th2 response of fetal lymphocytes. After delivery, while healthy infants switch their Th2 to Th1 profile by stimulation of infectious agents, this reversal does not occur in atopic individuals [23]. Their immunological reaction still favors the Th2 type which reacts to stimulants and results in allergic disease [24]. Smaller family, improved hygienic strategies, antibiotic usage etc all lead to reduction of surrounding microbes and supports this development [2].

In oriental medicine principles, eczema is considered as a systemic disease with imbalances of Qi or internal energy [14]. Many Asian patients would think that western medicine cannot offer a cure, and that topical steroids and associated treatment have significant side effects [13]. These principles all favor the concept that eczema is more like a systemic disease (atopy) with early skin manifestations (dermatitis) than a skin disease with systemic associations [5]. Recent research suggested that neutrophils, lymphocytes, eosinophils, immunoglobulins and complements are all important players in the pathophysiology of eczema [25]. It follows that treating eczema as a primary dermatological disease with only topical armamentarium without considering managing this complicated disease with a systemic and holistic approach is bound to meet with suboptimal effects [26]. Its clinical relevance and research interests are definitely beyond that of a mere cutaneous disease.

## **Competing interests**

The authors declare that they have no competing interests.

## Authors’ contributions

VY carried out the Pubmed search. KLH provided guidance about the search, performed the statistical analysis, and drafted the manuscript. TFL was responsible for reviewing and editing of the manuscript. All authors read and approved the final manuscript.
